# CC-99677, a novel, oral, selective covalent MK2 inhibitor, sustainably reduces pro-inflammatory cytokine production

**DOI:** 10.1186/s13075-022-02850-6

**Published:** 2022-08-18

**Authors:** Rajula Gaur, Kofi A. Mensah, Jason Stricker, Mary Adams, Anastasia Parton, Dorota Cedzik, Jamie Connarn, Michael Thomas, Gerald Horan, Peter Schafer, Stuart Mair, Maria Palmisano, Francisco Ramírez-Valle

**Affiliations:** 1 Bristol Myers Squibb, Princeton, NJ USA; 2 Quotient Sciences, Nottingham, UK

**Keywords:** Spondyloarthritis, Inflammatory arthritis, Mitogen-activated protein kinase kinases, Cytokines, Immunology, Clinical pharmacology

## Abstract

**Background:**

Mitogen-activated protein kinase (MAPK)-activated protein kinase-2 (MK2) is activated downstream of p38 MAPK and regulates stability of mRNAs encoding inflammatory cytokines. CC-99677 is a novel, irreversible, covalent MK2 inhibitor under development for the treatment of ankylosing spondylitis (AS) and other inflammatory diseases. As part of a phase I clinical trial to assess safety and tolerability, we evaluated target engagement, pharmacokinetics, and pharmacodynamics of CC-99677.

**Methods:**

The MK2 inhibitor CC-99677 was evaluated for its effect on cytokine expression in vitro in peripheral blood mononuclear cells (PBMCs) from healthy donors and patients with a definitive AS diagnosis. A novel in vitro model was developed to compare the potential for tachyphylaxis of CC-99677 and p38 inhibitors in THP-1 cells. The effect of CC-99677 on tristetraprolin (TTP) and cytokine mRNA was assessed in stimulated human monocyte-derived macrophages. In a first-in-human study, thirty-seven healthy volunteers were randomly assigned to daily oral doses of CC-99677 or placebo, and blood was collected at pre-specified time points before and after dosing. CC-99677 concentrations were assessed in the plasma, and CC-99677 binding to MK2 was evaluated in PBMCs. Ex vivo stimulation of the whole blood was conducted from participants in the first-in-human study to assess the pharmacodynamic effects.

**Results:**

In vitro, CC-99677 inhibited tumor necrosis factor (TNF), interleukin (IL)-6, and IL-17 protein production in samples of monocytes and macrophages from AS patients and healthy volunteers via an mRNA-destabilization mechanism. In the in vitro model of tachyphylaxis, CC-99677 showed a differentiated pattern of sustained TNF protein inhibition compared with p38 inhibitors. CC-99677 reduced TTP phosphorylation and accelerated the decay of inflammatory cytokine mRNA in lipopolysaccharide-stimulated macrophages. Administration of CC-99677 to healthy volunteers was safe and well-tolerated, with linear pharmacokinetics and sustained reduction of ex vivo whole blood TNF, IL-6, and chemokine synthesis.

**Conclusions:**

CC-99677 inhibition of MK2 is a promising approach for the treatment of inflammatory diseases and may overcome the limitations of p38 MAPK inhibition.

**Trial registration:**

ClinicalTrials.gov NCT03554993.

**Supplementary Information:**

The online version contains supplementary material available at 10.1186/s13075-022-02850-6.

## Background

Spondyloarthritis comprises a group of inflammatory rheumatic diseases that is characterized by spinal and peripheral joint oligoarthritis and enthesitis and may be associated with mucocutaneous, ocular, and/or cardiac comorbidities [1]. Treatment for these diseases consists of nonsteroidal anti-inflammatory drugs as the first line of therapy, with biologic agents targeting cytokines, such as tumor necrosis factor (TNF) and interleukin (IL)-17, considered for patients who do not respond [2]. Although TNF and IL-17 inhibitors have demonstrated improved disease-related parameters in patients with ankylosing spondylitis (AS), the effects on radiographic progression are less well defined [3, 4]. Therefore, an unmet need exists for new treatments that address both disease-related parameters and radiographic progression.

The p38–mitogen-activated protein kinase (MAPK) signaling pathway plays a central role in the stress-mediated production of proinflammatory cytokines (TNF, IL-6, and IL-1β), and inhibitors of p38-MAPK have been extensively investigated for the treatment of inflammatory diseases [5–7]. p38-MAPK has nearly one hundred known substrates, including kinases, transcriptional factors and regulators, cell cycle regulators, and other MAPKs [8]. Inhibitors of p38-MAPK with autoimmune disease-related indications have failed to advance in clinical development, due to hepatotoxicity, cardiotoxicity, and lack of efficacy [5, 7, 9, 10].

p38-MAPK inhibitors have been associated with tachyphylaxis in patients with rheumatoid arthritis and other inflammatory diseases, in which early reduction in inflammatory markers such as C-reactive protein (CRP) did not persist despite continued treatment [5, 9, 11]. The observation of tachyphylaxis may be explained by the pleiotropic role of p38, and inhibition of p38-MAPK may result in the activation of compensatory mechanisms. For instance, p38 inhibition has been observed to activate the upstream kinases MAPK kinase 4 and 7 (MKK4/7) and inhibit the negative MAPK regulator dual-specificity phosphatase 1 (DUSP1), which inhibits multiple MAPKs including p38-MAPK [12, 13]. Therefore, as a means to avoid the observed limitations inherent in targeting p38-MAPK, proteins downstream of p38-MAPK have been identified as new targets for inflammatory diseases.

MAPK-activated protein kinase-2 (MK2), a direct downstream target of p38, has been identified as a promising target for inflammatory diseases. Activation of MK2 increases the stability and translation of mRNA of proinflammatory factors (e.g., TNF, IL-1β, IL-6) [14–16]. The effect of MK2 at the post-transcriptional level is mediated by tristetraprolin (TTP), a zinc finger binding protein that binds to the AU-rich elements (ARE) in the 3′ untranslated regions (UTRs) [17]. Through the recruitment of deadenylases, TTP destabilizes the mRNA of multiple cytokines containing ARE in their 3′UTRs, including TNF, GM-CSF, IL-10, IL-6, and also its own mRNA, resulting in an auto-regulatory negative feedback loop [18–20]. Human antigen R (HuR), an RNA-stabilizing protein, plays an opposing role to TTP by competing for binding to the ARE and promoting translation. Activated MK2 phosphorylates TTP and reduces its affinity to ARE, thereby allowing HuR to bind and promote cytokine production by stabilizing mRNA and enhancing translation [17]. MK2 knockout mice, unable to stabilize transcripts of pro-inflammatory cytokines via TTP, are protected from inflammation in various inflammatory disease models [15, 21]. MK2 inhibitors have demonstrated efficacy in models of arthritis and AS [21–23].

Here, we describe in vitro studies using peripheral blood mononuclear cells (PBMCs) from AS patients and healthy volunteers to analyze the effect of CC-99677 on proinflammatory proteins. To delineate the mechanism of action of CC-99677 on cytokine production, we investigated its effect on TTP phosphorylation and on cytokine mRNA decay in macrophages. We explore the relationship between drug exposure and tachyphylaxis potential in a novel in vitro assay in THP-1 cells and contrast CC-99677 activity with activity of p38 inhibitors. We also report data from the phase I first-in-human (FIH) multiple ascending-dose (MAD) study to characterize the safety, pharmacokinetics (PK), and pharmacodynamics (PD) of CC-99677 in healthy volunteers treated with CC-99677; single ascending-dose data are reported elsewhere [23, 24].

## Methods

### Culturing PBMCs from healthy and AS patient samples

Human PBMCs from healthy volunteers and patients with AS from Bio-Options (Brea, CA) were thawed and washed with phosphate-buffered saline (PBS). For lipopolysaccharide (LPS) stimulation, RPMI 1640 medium, supplemented with 10% fetal bovine serum (FBS) and penicillin/streptomycin, was used to culture cells. Cells were pretreated with dimethyl sulfoxide (DMSO; 0.25%) or CC-99677 at 0.3, 1, and 3 μM for 1 h followed by LPS at 50 ng/mL for 24 h. In the case of Staphylococcal enterotoxin-B (SEB)/IL-2 treatment, cells were cultured in RPMI 1640 medium, supplemented with 10% FBS, 1% GlutaMAX, and 1% HEPES and penicillin/streptomycin, for 3 days. All cells were cultured at 37 °C with 5% CO_2_ in a humidified incubator. The culture media were harvested and frozen for cytokine and chemokine assessment.

### Daily treatment of THP-1 cells with CC-99677 or p38 inhibitors

THP-1 cells were seeded at 1.0 × 10^6^ cells/mL and allowed to acclimate for 24 h. Cell count and viability were measured daily to monitor cell number and health. To maintain the cell populations around 1.0 × 10^6^ cells/mL, cells were split every other day. p38 inhibitors (BMS-582949, BIRB-796, and SCIO-469), MK2 inhibitor CC-99677, or DMSO were added daily to maintain constant exposure until the time of sample collection. Samples were collected on days 0, 1, 3, 6, 9, 12, and 14. At each time point, the cells were stimulated with 100 ng/mL LPS for 24 h, to mimic the ex vivo assay that is performed in the clinic, and TNF levels were measured in the supernatants using enzyme-linked immunosorbent assay (ELISA; cat. no. 18421, Abcam, Cambridge, MA).

### Cytokine analysis by ELISA

Culture supernatants were centrifuged, and the supernatants were collected and analyzed for selected cytokines and chemokines using Milliplex Magpix assays (catalog HCYTO – 60K; Millipore Sigma, Burlington, MA). Levels of proinflammatory cytokines TNF and MCP-1 for the monocytes and macrophages were quantified using specific ELISA kits (R&D, NJ, USA) according to the manufacturer’s recommendations. Data were processed using Milliplex Analyst, and significance was calculated using the one-way analysis of variance (ANOVA) with Dunnett’s post-test algorithms in GraphPad Prism version 5.01.

### Differentiating human monocytes into macrophages

PBMCs were isolated from buffy coat using the Ficoll gradient method. Monocytes were isolated using the STEMCELL Technologies Inc. kit (catalog number 19058; Vancouver, BC, Canada). Monocytes were differentiated into macrophages by stimulating with 50 ng/mL of macrophage colony-stimulating factor (M-CSF) for 5 days followed by treatment with 50 ng/mL interferon gamma (IFNγ) for ~ 16 h. Macrophages were then plated and allowed to acclimate for 24 h.

### Assessing the effect of CC-99677 on cytokines and TTP

Monocytes and macrophages were pre-treated for 1 h with DMSO or compound, followed by LPS stimulation (100 ng/mL) for 4 or 24 h (includes 1 h pre-treatment). Medium from the cultured macrophages was used for cytokine analysis using Magpix magnetic beads (Catalog HCYTO – 60K; Millipore Sigma, Burlington, MA). Cell pellets from the macrophage cultures were collected for western blot analysis using capillary electrophoresis to analyze the effect on TTP, with glyceraldehyde 3-phosphate dehydrogenase (*GAPDH*) used as the house-keeping protein in the capillary electrophoresis. Antibodies against total TTP (CST#7162 Rabbit Monoclonal antibody) and GAPDH (CST#5174 Rabbit Monoclonal antibody) were used to detect the respective proteins.

### Assessing mRNA stability in macrophages

Monocytes differentiated into macrophages with M-CSF and IFNγ were stimulated with LPS for 3 h followed by treatment with 10 μg/mL actinomycin D in the presence of 1 μM CC-99677 or DMSO for 0 h, 10 min, 30 min, and 1, 2, 4, 5, and 6 h at 37 °C. After each time point, macrophages were washed with PBS and lysed with RLT Plus Lysis Buffer (Qiagen, Germantown, MD). RNA was isolated using the QiaCube, followed by cDNA preparation using Superscript IV enzyme mix (Invitrogen, Thermo Fisher, Waltham, MA). Rate of mRNA decay by actinomycin D and CC-99677 was monitored by reverse transcription polymerase chain reaction with human primers against *TNF*, *IL-6*, and *IL-1β* using *GAPDH* as a housekeeping gene.

### Clinical study design

A single-center, randomized, double-blind, placebo-controlled phase I study (NCT03554993) with a MAD component was performed in the UK between May 2018 and July 2019. Thirty-seven healthy volunteers of any race, ethnicity, or sex were enrolled. The inclusion criteria were 18 to 55 years of age inclusive, body mass index 18 to 33 kg/m^2^, and physical examination by the investigator without clinically significant findings. Subjects were excluded if any clinically significant medical history, laboratory abnormality, psychiatric illness, or other condition existed that would place the subject at unacceptable risk by participating in the study. Pregnancy prevention guidance was provided as per Clinical Trial Facilitation Group recommendations [25]. Subjects were randomly assigned to daily oral doses of CC-99677 or placebo. Subjects were dosed daily for 14 days. There were 5 dose-escalation cohorts ranging from 10 to 150 mg of CC-99677 or placebo. Both the investigator and subjects were blinded to the treatment arm. Safety assessments, including physical examinations, clinical laboratory tests, and collection of adverse events (AEs) were performed and graded using MedDRA version 22.0 and the US Food and Drug Administration (FDA) Toxicity Grading Scale for Healthy Adult and Adolescent Volunteers [26]. The primary outcome of this trial was the safety and tolerability of multiple daily doses of CC-99677. Secondary and exploratory outcomes included assessment of the PK profile and evaluation of the PD effects of CC-99677 as measured by MK2 occupancy and ex vivo inhibition of inflammatory cytokines.

### Target engagement assay

Target engagement of MK2 was measured using the streptavidin mass shift (SMaSh) assay in PBMCs, which is designed to indicate amounts of MK2 occupied and unoccupied by covalently bound CC-99677 [23, 27]. Blood was collected for PBMC isolation at pre-specified time points, before dosing on days 1, 2, 3, 5, 7, and 14 and then following the last dose on days 15, 17, 21, and 28. Samples were evaluated for the percentage of MK2 bound to CC-99677 versus the percentage of free MK2 in PBMCs under the indicated cohort dosage conditions. Target engagement was measured by calculating the change in percentage bound MK2 from baseline.

### Pharmacokinetic assessment

Blood was collected for the determination of CC-99677 plasma concentrations at pre-specified time points for intense PK sampling. Concentrations of CC-99677 in the plasma were measured using a validated liquid chromatography tandem mass spectrometry assay. PK parameters were calculated via non-compartmental methodology using Phoenix version 7 or higher (Princeton, NJ).

### Pharmacodynamic assessment

Effects of CC-99677 on innate cell activities were evaluated by assessing cytokine and chemokine production following LPS stimulation ex vivo. Ex vivo stimulation was performed in whole blood samples collected before dosing on days 1, 2, 3, 5, 7, and 14 and then following the last dose on days 15, 17, 21, and 28 using the TruCulture® assay system (Myriad RBM, Austin, TX). Briefly, blood (1 mL) was drawn into the LPS-containing TruCulture tubes. The tubes were placed in a 37 °C block thermostat for 24 ± 1 h and then frozen at −70 °C. Media mixed with plasma were analyzed for TNF and other cytokine/chemokine levels. TNF inhibition in ex vivo LPS-stimulated blood was measured as a percentage change in TNF levels from baseline. Percentage change in other cytokines and chemokines in the ex vivo stimulation assay was also measured with respect to their baseline levels.

## Results

### CC-99677 inhibited cytokine production in PBMCs from patients with AS

**Fig. 1:**
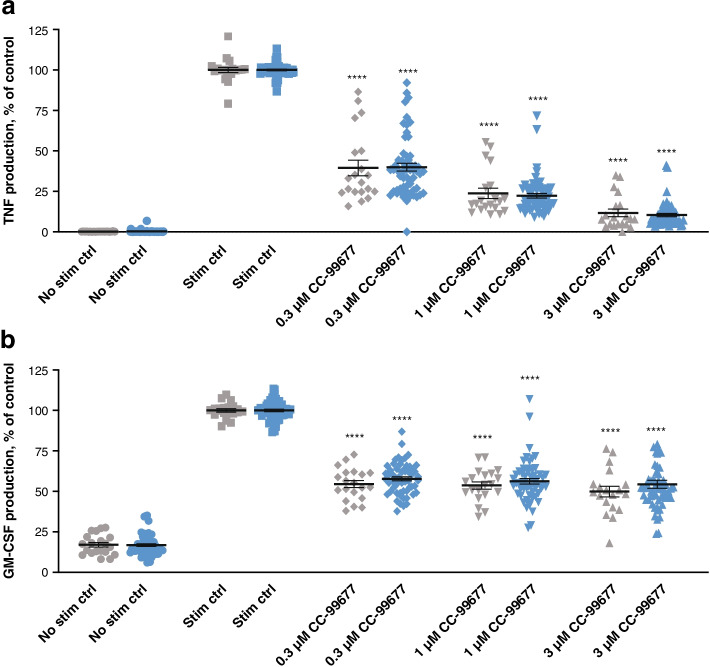
CC-99677 showed concentration-dependent inhibition of TNF and GM-CSF production in LPS-stimulated PBMCs. TNF (**a**) and GM-CSF (**b**) inhibition in LPS-stimulated PBMCs from patients with AS and healthy volunteers. PBMCs were treated with CC-99677 for 1 h followed by LPS stimulation for 18 h. Magnetic beads were used to assess TNF and GM-CSF. Each sample was analyzed in duplicate, and data were normalized to the stimulation control (100%). The mean percent inhibition is indicated by a horizontal line with error bars for the standard error of the mean; significance was calculated using the one-way ANOVA with Dunnett’s post-test algorithms in GraphPad Prism version 5.01. Gray symbols indicate the data points from healthy donors (*n* = 10), and blue symbols indicate the data points from donors with AS (*n* = 30). ANOVA, analysis of variance; AS, ankylosing spondylitis; GM-CSF, granulocyte-macrophage colony-stimulating factor; IL, interleukin; LPS, lipopolysaccharide; MCP-1, monocyte chemoattractant protein 1; PBMC, peripheral blood mononuclear cell; stim ctrl, stimulation control; TNF, tumor necrosis factor. ****p < 0.0001; ***p < 0.001; **p < 0.01; *p < 0.05

We have previously shown that CC-99677 inhibits inflammatory cytokine production from LPS-stimulated healthy donor PBMCs, including TNF, IL-6, and granulocyte-macrophage colony-stimulating factor (GM-CSF) [23]. We investigated the effect of CC-99677 in AS patient samples and observed a similar concentration-dependent inhibition of TNF production in LPS-stimulated PBMCs (Fig. 1A). Because GM-CSF may prime PBMCs and monocytes from patients with AS for increased TNF production, we also determined the effect of CC-99677 on GM-CSF production [28, 29]. CC-99677 inhibited GM-CSF in PBMCs from AS patients to similar levels as those seen in healthy volunteers (Fig. 1B).

**Fig. 2:**
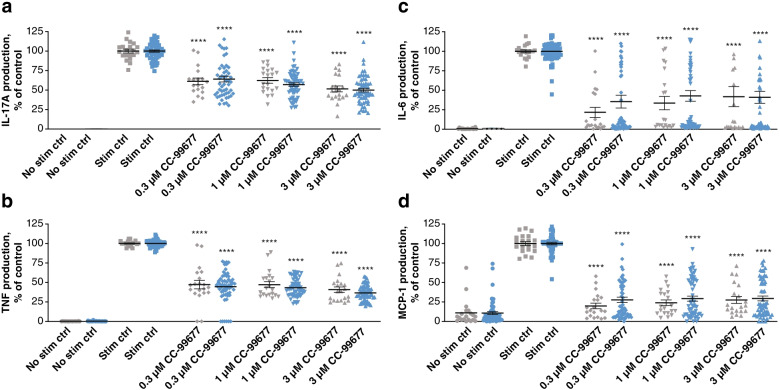
CC-99677 inhibited the production of TNF, IL-6, IL-17A, and MCP-1 from SEB-1/IL-2-stimulated human PBMCs. TNF (**a**), IL-6 (**b**), IL-17A (**c**), and MCP-1 (**d**) inhibition in SEB/IL-2-stimulated PBMCs from patients with AS and healthy volunteers. PBMCs were treated with CC-99677 for 1 h followed by SEB + IL-2 stimulation for 72 h. Magnetic beads were used to assess TNF, IL-6, IL-17A, and MCP-1. Each sample was analyzed in duplicate, and data were normalized to the stimulation control (100%). The mean percent inhibition is indicated by a horizontal line with error bars for the standard error of the mean; significance was calculated using the one-way ANOVA with Dunnett’s post-test algorithms in GraphPad Prism version 5.01. Gray symbols indicate the data points from healthy donors (*n* = 10), and blue symbols indicate the data points from donors with AS (*n* = 30). ANOVA, analysis of variance; AS, ankylosing spondylitis; IL, interleukin; MCP-1, monocyte chemoattractant protein 1; PBMC, peripheral blood mononuclear cell; SEB, Staphylococcal enterotoxin B; stim ctrl, stimulation control; TNF, tumor necrosis factor. ****p < 0.0001; ***p < 0.001; **p < 0.01; *p < 0.05

To extend these findings, we stimulated PBMCs with SEB and IL-2 to determine the effect of cytokine production downstream of T-cell activation. In this model, CC-99677 inhibited TNF, IL-17A, IL-6, and monocyte chemoattractant protein-1 (MCP-1) expression in PBMCs from both healthy donors and AS patients (Fig. 2).

### MK2 inhibition reduced cytokine production in LPS-stimulated monocytes and macrophages

**Fig. 3:**
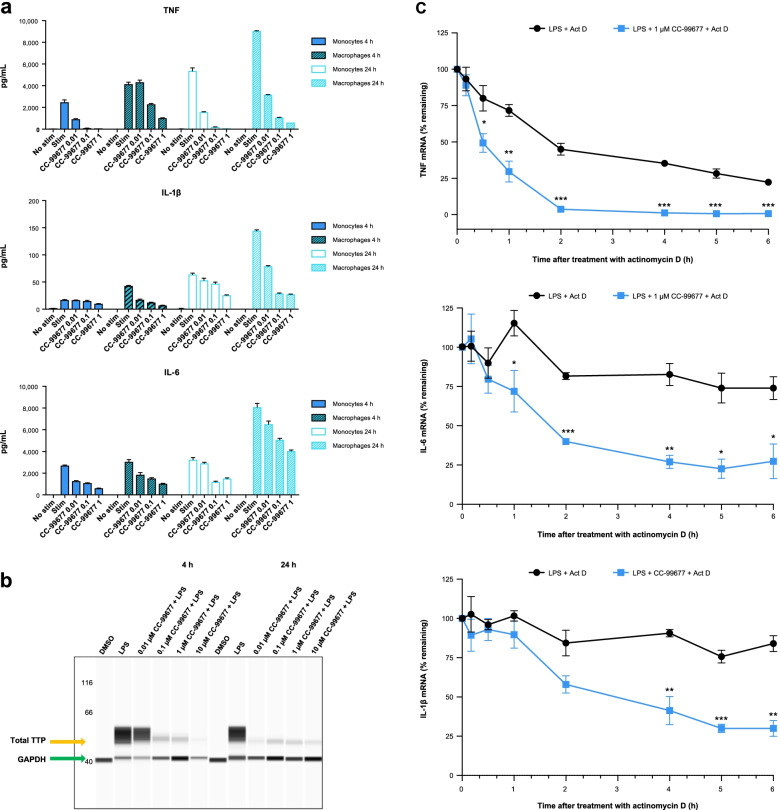
CC-99677 inhibited the cytokine production in monocytes and macrophages and reduced the TTP expression through inhibition of MK2. Human monocytes (*n* = 3) were isolated from the whole blood and were differentiated into macrophages using M-CSF followed by IFNγ. Both monocytes and macrophages were treated with the depicted micromolar concentrations of CC-99677 for 1 h, and the cytokines shown were analyzed after stimulation with 100 ng/mL LPS for 4 or 24 h (**a**). Lysates from macrophages pretreated with CC-99677 followed by LPS stimulation for 4 h or 24 h were analyzed by capillary electrophoresis and probed with total TTP antibody and GAPDH antibody (**b**). *TNF*, *IL-6*, and *IL-1β* transcripts were measured at depicted times in macrophages stimulated with LPS and then treated with actinomycin D in the presence of CC-99677 or DMSO (**c**). Act D, actinomycin D; AUC, area under the curve; DMSO, dimethylsulfoxide; GAPDH, glyceraldehyde 3-phosphate dehydrogenase; IL, interleukin; IFNγ, interferon gamma; LPS, lipopolysaccharide; M-CSF, macrophage colony-stimulating factor; MK2, mitogen-activated protein kinase-activated protein kinase-2; TNF, tumor necrosis factor; TTP, tristetraprolin

Myeloid cells such as monocytes are critical producers of TNF, IL-23, and other inflammatory cytokines in AS [30, 31]. Given their key pro-inflammatory roles, we sought to determine the effect of CC-99677 on LPS-stimulated monocytes and macrophages. Macrophages were derived from monocytes and differentiated into pro-inflammatory M1 type by M-CSF treatment followed by IFNγ. CC-99677 exhibited strong concentration-dependent inhibition of TNF in both monocytes and macrophages (Fig. 3A). CC-99677 also exhibited concentration-dependent inhibition of IL-6 and IL-1β secretion in monocytes and macrophages, although the inhibitory effects of CC-99677 on cytokine secretion were more potent in macrophages (Fig. 3A).

### CC-99677 prevented TTP phosphorylation to promote cytokine mRNA decay

To confirm the mechanism of cytokine inhibition downstream of MK2 inhibition, we evaluated the effect of CC-99677 on the TTP phosphorylation state and cytokine mRNA stability. We used an antibody against TTP to investigate the effect on protein levels by capillary electrophoresis. Although phospho-TTP-specific antibodies are not available, total TTP protein levels can be used as a surrogate for the TTP phosphorylation state. The stability and total abundance of TTP are regulated by its phosphorylation by MK2: when TTP is dephosphorylated, it becomes unstable and therefore total TTP protein levels are reduced [20]. As expected, LPS stimulation in macrophages resulted in TTP phosphorylation reflected by increased TTP abundance (Fig. 3B). Notably, CC-99677 treatment resulted in a dose-dependent reduction in total TTP levels, indicating a reduced phosphorylation state. We then assessed the effect of CC-99677 inhibition of TTP phosphorylation on cytokine mRNA stability. The mRNA decay of ARE-containing *TNF*, *IL-6*, and *IL-1β* transcripts was measured in LPS-stimulated macrophages in the presence of actinomycin D alone and in combination with CC-99677. The expression of each pro-inflammatory gene transcript was normalized to *GAPDH* mRNA. A decrease in cytokine mRNA levels was observed with actinomycin D alone over time; however, the combination of actinomycin D and CC-99677 resulted in significantly greater mRNA decay compared with actinomycin D alone (Fig. 3C).

### Sustained in vitro TNF inhibition by CC-99677 but not by p38 inhibitors

**Fig. 4:**
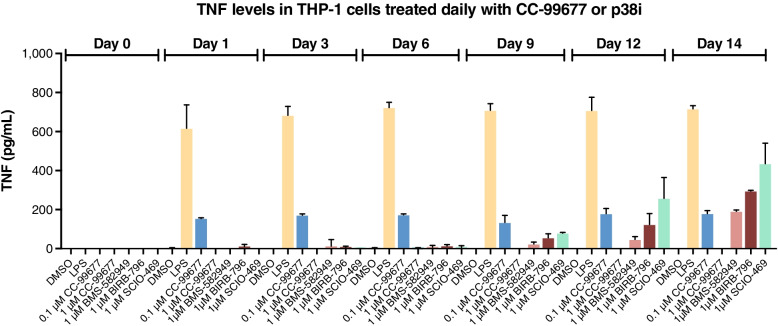
Sustained inhibition of TNF in THP-1 cells continually exposed to CC-99677. Effect of continuous inhibition of MK2i (CC-99677) or p38i (BMS-582949, BIRB-796, SCIO-794) on TNF secretion in LPS-stimulated THP-1 cells. Cells were treated with 0.1 or 1 μM CC-99677 or 1 μM p38 inhibitors for 23 h and then stimulated with LPS for 24 h. TNF secreted into the culture media was determined using magnetic beads. Data are mean (SD). LPS, lipopolysaccharide; MK2i, mitogen-activated protein kinase-activated protein kinase-2 inhibitor; p38i, p38 inhibitor; SD, standard deviation; TNF, tumor necrosis factor

In clinical studies with p38 inhibitors, early reduction in proinflammatory markers such as CRP was not sustained over the treatment period. Inflammatory marker levels were found to rebound as early as 2 weeks after treatment, suggesting tachyphylaxis [5, 7, 9, 10]. We sought to develop a model of continued inhibitor treatment to determine the potential for tachyphylaxis in vitro. THP-1 cells were treated daily with p38 inhibitors or CC-99677 for up to 14 days. At different times during this treatment period, an aliquot of cells was stimulated with LPS. As expected, at early times during the treatment period, p38 inhibitors blocked LPS-stimulated TNF production (Fig. 4). However, beginning on day 9, loss of TNF inhibitory effect was noted for all p38 inhibitors tested (BMS-582949, BIRB-796, SCIO-469). In contrast to p38 inhibitors, CC-99677 suppressed TNF production throughout the treatment period. These data suggest that MK2 inhibition may circumvent tachyphylaxis associated with p38 inhibitors.

### CC-99677 was safe and well-tolerated in a multiple ascending dose study

The effects of CC-99677 in vitro and in vivo supported the initiation of a FIH clinical study in healthy volunteers [23]. The PK and PD of single ascending doses of CC-99677 have been reported [23]. The results from the MAD portion of the study are reported here. Thirty-seven healthy adults (67.6% male, 32.4% female, 89.2% white) with an average age of 34 years (range 20–55) and average body mass index of 26.08 kg/m^2^ (range 20–33) completed the once-daily multiple-dosing study. Aside from a slight gender imbalance, demographics were comparable between subjects dosed with CC-99677 and those with placebo (Additional file 1: Table S1).

After 14 consecutive days of dosing, no serious treatment-emergent adverse events (TEAEs) were observed in any of the 37 subjects among the 5 dose-level cohorts ranging from 10 to 150 mg of CC-99677. No subject discontinued the MAD study because of safety reasons. All TEAEs in the MAD study were graded by the investigator as mild (grade 1) according to the FDA toxicity grading scale [26]. The complete TEAE listing is shown in Table 1. TEAEs occurred in 77.8% of subjects receiving placebo compared with 46.4% of subjects receiving CC-99677 (Table 1; Fig. S1A). There was no apparent CC-99677 dose dependency observed with respect to the incidence of TEAEs (Table 1; Fig. S1B). The most commonly reported TEAE was mild headache (*n* = 5), followed by mild myalgia (*n* = 3) (Table 1; Fig. S1C). Causality assessment was performed in a blinded fashion; the suspected relationship of TEAE to study drug, as assessed by the investigator, showed that the majority (69%) of AEs were not suspected to be related to CC-99677 (Fig. S1D). There were no trends among the AEs suspected of relationship to the study drug.

**Table 1:** Treatment-emergent adverse events by system organ class

Treatment-emergent AE, ***n*** (%)	CC-99677 10 mg (***n*** = 5)	CC-99677 30 mg (***n*** = 6)	CC-99677 60 mg (***n*** = 6)	CC-99677 120 mg (***n*** = 6)	CC-99677 150 mg (***n*** = 5)	Placebo (***n*** = 9)
Subjects with ≥ 1 treatment-emergent AE	3 (60.0)	4 (66.7)	1 (16.7)	3 (50.0)	2 (40.0)	7 (77.8)
Nervous system disorders	2 (40.0)	1 (16.7)	1 (16.7)	1 (16.7)	0	1 (11.1)
Headache	2 (40.0)	0	1 (16.7)	1 (16.7)	0	1 (11.1)
Dizziness	1 (20.0)	1 (16.7)	0	0	0	0
Gastrointestinal disorders	2 (40.0)	2 (33.3)	0	1 (16.7)	0	2 (22.2)
Nausea	2 (40.0)	0	0	0	0	0
Constipation	0	0	0	0	0	2 (22.2)
Diarrhea	0	0	0	1 (16.7)	0	0
Abdominal pain	0	0	0	1 (16.7)	0	0
Chapped lips	0	1 (16.7)	0	0	0	0
Dyspepsia	1 (20.0)	0	0	0	0	0
Rectal hemorrhage	0	1 (16.7)	0	0	0	0
Musculoskeletal and connective tissue disorders	0	2 (33.3)	0	1 (16.7)	0	2 (22.2)
Back pain	0	0	0	0	0	1 (11.1)
Myalgia	0	2 (33.3)	0	0	0	1 (11.1)
Musculoskeletal pain	0	0	0	1 (16.7)	0	0
Infections and infestations	1 (20.0)	1 (16.7)	0	0	0	1 (11.1)
Nasopharyngitis	0	1 (16.7)	0	0	0	1 (11.1)
Rash pustular	1 (20.0)	0	0	0	0	0
General disorders and administration site conditions	2 (40.0)	0	0	0	0	2 (22.2)
Vessel puncture site pain	1 (20.0)	0	0	0	0	0
Catheter site related reaction	0	0	0	0	0	1 (11.1)
Sensation of foreign body	1 (20.0)	0	0	0	0	0
Vessel puncture site reaction	0	0	0	0	0	1 (11.1)
Respiratory, thoracic, and mediastinal disorders	1 (20.0)	0	0	1 (16.7)	0	0
Oropharyngeal pain	1 (20.0)	0	0	1 (16.7)	0	0
Injury, poisoning, and procedural complications	1 (20.0)	0	0	0	0	0
Muscle strain	1 (20.0)	0	0	0	0	0
Investigations	0	0	0	1 (16.7)	1 (20.0)	0
Alanine aminotransferase increased	0	0	0	1 (16.7)	0	0
Aspartate aminotransferase increased	0	0	0	0	1 (20.0)^a^	0
Blood creatine phosphokinase increased	0	0	0	0	1 (20.0)^a^	0
Vascular disorders	0	0	0	0	1 (20.0)	1 (11.1)
Flushing	0	0	0	0	1 (20.0)	0
Hot flush	0	0	0	0	0	1 (11.1)
Ear and labyrinth disorders	1 (20.0)	0	0	0	0	0
Ear pain	1 (20.0)	0	0	0	0	0
Psychiatric disorders	1 (20.0)	0	0	0	0	0
Insomnia	1 (20.0)	0	0	0	0	0
Reproductive system and breast disorders	0	0	0	0	1 (20.0)	0
Dysmenorrhea	0	0	0	0	1 (20.0)	0

*AE* adverse event

^a^Occurred in the same individual 14 days after the end of dosing following greater-than-usual physical activity by the individual

### CC-99677 exhibited a linear and predictable PK profile

The multiple-dose PK characteristics of CC-99677 showed a linear, dose-proportional increase in concentration and exposure from 10 to 150 mg with a once-daily dosing schedule over 14 days. The geometric mean peak concentration (*C*_max_, CV%) on day 1 was 20.5 (51.4%), 63.4 (22.1%), 150 (66.4%), 269 (44.7%), and 257 (37.1%) ng/mL, at 10, 30, 60, 120, and 150 mg, respectively. At all 5 dose levels, the PK profile showed minimal detectable plasma CC-99677 > 24 h after dosing, which indicates limited to no day-to-day plasma accumulation of CC-99677 [23]. In line with this finding, no significant difference was observed between (1) dosing day 1 peak or total exposure and (2) peak and total exposure when target engagement steady-state was achieved [32]. The geometric mean *C*_max_ at the 150-mg dose level corresponds to approximately the 0.8 μM concentration of CC-99677. This concentration falls in the range where significant inhibition of TNF and other cytokines was seen in preclinical in vitro assays.

### MK2 target engagement in PBMCs of healthy volunteers dosed with CC-99677

**Fig. 5:**
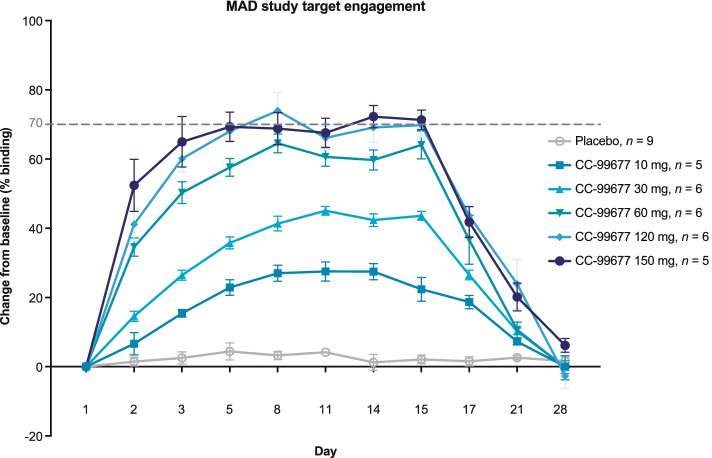
Covalently bound CC-99677 resulted in a cumulative increase in MK2 occupancy with daily dosing. CC-99677 target engagement with MK2 binding measured as a percentage change from baseline with the last dose on day 14. Measurements in PBMCs isolated from the whole blood of healthy subjects dosed with CC-99677 in MAD study with streptavidin mass shift assay. Data depicted as means with standard error of the means. MAD, multiple ascending dose; MK2, mitogen-activated protein kinase-activated protein kinase-2; PBMC, peripheral blood mononuclear cell

The SMaSh assay, which differentiated the levels of free MK2 and CC-99677-bound MK2, demonstrated time- and dose-dependent covalent binding of CC-99677 in lysates of PBMCs from healthy volunteers dosed orally with CC-99677 (Fig. 5). CC-99677 demonstrated dose-dependent increases in MK2 occupancy between 10 and 120 mg, with a plateau at the 120- and 150-mg dose levels. The average MK2 occupancy increased from 28% at day 8 in the 10-mg dose cohort to approximately 70% in the 120- and 150-mg dose cohorts (Fig. 5). MK2 occupancy by CC-99677 was stable until day 15 (1 day after the last dose) and returned to baseline levels 14 days after the last dose.

**Fig. 6:**
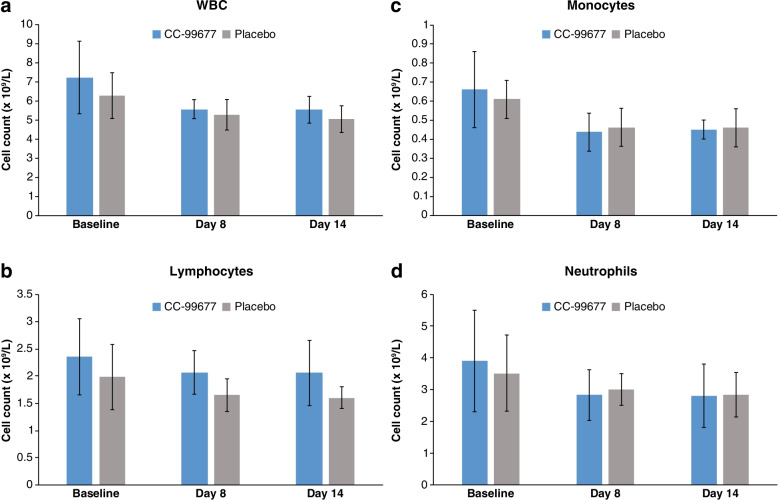
Maximal target engagement with daily CC-99677 dosing does not change leukocyte/leukocyte subtype cell counts. Total WBC count (**a**), lymphocyte count (**b**), monocyte count (**c**), and absolute neutrophil count (ANC) (**d**) are shown at the indicated time points of dosing. (*Note*: neutropenia defined as ANC < 1.5 × 10^9^/L). All CC-99677 data at 150-mg dose level from pre-dose baseline to last dose on day 14 (*n* = 5). Total placebo *n* = 9. Data are mean (SD). SD, standard deviation; WBC, white blood cell

Having demonstrated a cumulative increase in target engagement with daily dosing, we sought to evaluate the effect on cytokine production in ex vivo peripheral blood stimulation assays. To ensure that cytokine changes were not potentially due to changes in peripheral blood cell numbers induced by dosing with CC-99677, we compared total leukocyte, monocyte, lymphocyte, and neutrophil counts between active and placebo cohorts. Total white blood cell (WBC), lymphocyte, monocyte, and neutrophil counts were monitored from pre-dose baseline through the 14-day daily dosing period. At 150 mg CC-99677, where > 70% MK2 target engagement was observed, the mean (standard deviation [SD]) WBC, lymphocyte, monocyte, and neutrophil counts were comparable to placebo, and no subject had an absolute neutrophil count (ANC) diagnostic of neutropenia (ANC < 1.5 × 10^9^ cells/L) (Fig. 6 A–D).

### CC-99677 administration results in sustained inhibition of ex vivo LPS-stimulated cytokine production in healthy volunteers

**Fig. 7:**
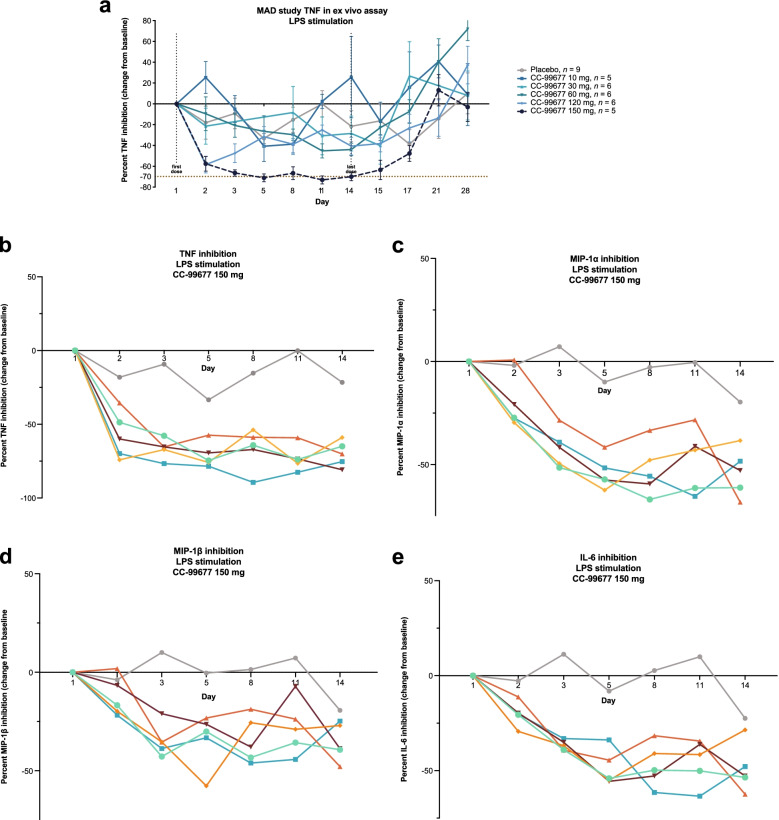
CC-99677 administration resulted in sustained inhibition of ex vivo LPS-stimulated cytokine production in healthy volunteers. TNF levels (**a**) as percentage change from baseline measured in ex vivo LPS-stimulated whole blood from heathy volunteers until day 28 (lines show mean data for each group). TNF (**b**), MIP-1α (**c**), MIP-1β (**d**), and IL-6 (**e**) levels as percentage change from baseline measured in ex vivo LPS-stimulated whole blood from heathy volunteers dosed with 150 mg of CC-99677 (gray line represents the mean percent change in cytokine levels of 9 volunteers who received placebo; colored lines represent the percent change in cytokine level for individual CC-99677-dosed subjects; data shown until last day of dosing, day 14). Data calculated as the median percentage change from baseline. IL, interleukin; LPS, lipopolysaccharide; MIP, macrophage inflammatory protein; TNF, tumor necrosis factor

CC-99677 inhibited TNF at all doses > 10 mg, and this inhibition was sustained until day 14 (Fig. 7A). TNF production was reduced as early as day 2 in the 60-, 120-, and 150-mg dose cohorts. Maximal TNF inhibition was 70% in the 150-mg dose cohort and was consistently maintained at approximately 70% during the dosing period (Fig. 7B). Other cytokines and chemokines were inhibited by CC-99677, but more variability was observed compared with TNF inhibition. Macrophage inflammatory protein (MIP)-1α and MIP-1β were inhibited with continuous dosing at 120 and 150 mg (Fig. 7 C, D). IL-6 production in particular demonstrated marked variability, which may reflect diurnal effects on IL-6 levels [33]. Despite this variability, IL-6 was clearly inhibited at the 150-mg dose level (Fig. 7E). Maximal inhibition of 50% was observed for IL-6 and MIP-1α in the 150-mg dose level cohort at day 5.

## Discussion

Inhibitors of p38 kinases have been explored as potential anti-inflammatory therapies, but their development was hindered by unfavorable safety profiles and evidence of tachyphylaxis [5, 9]. We have characterized the inhibitory profile of CC-99677 in PBMCs from patients with AS that were stimulated with LPS or SEB/IL-2 to assess the effect of MK2 inhibition in innate and adaptive immune cells in vitro. LPS stimulation of PBMCs focuses on assessing myeloid responses, whereas SEB stimulation focuses on the response elicited from antigen-presenting cells (APCs) and T-cells by binding to class II major histocompatibility complex proteins on APCs and T-cell receptors at the variable region of the β-chain [34]. CC-99677 inhibited TNF and GM-CSF in LPS-stimulated PBMCs and TNF, IL-6, IL-17A, and MCP-1 in SEB/IL-2-stimulated PBMCs. CC-99677 showed strong concentration-dependent inhibition of TNF, IL-6, and IL-1β cytokine production in M1-polarized macrophages and similar, albeit slightly less potent, effects in monocytes. The inhibition of IL-1β in macrophages and monocytes contrasts with the observation in PBMCs, where no inhibition of IL-1β was observed after LPS stimulation, suggesting pro-inflammatory macrophages derived from monocytes with M-CSF and IFNγ treatment have increased sensitivity to MK2 inhibition following LPS stimulation [23].

The constellation of cytokines and chemokines inhibited by CC-99677 may have translational relevance to inflammatory diseases including AS. TNF and IL-17A blockade are efficacious in the treatment of AS [2]. Circulating IL-6 levels are increased in patients with active AS, correlate with disease activity, and may predict treatment response to TNF inhibition [35, 36]. MCP-1 is elevated in the serum of patients with AS and may distinguish patients with mechanical low back pain from AS; furthermore, expression of MCP-1 and its receptor CC chemokine receptor 2 is increased in synovial samples from patients with AS [37, 38]. Additionally, the inhibitory effect in macrophages suggests that CC-99677 may be effective in suppressing inflammation in an inflamed environment where a significant proportion of pro-inflammatory cytokines are secreted by M-1 type macrophages [39]. Macrophages have been identified as predominant inflammatory cell types infiltrating entheseal sites in AS patient samples [40]. This supports the idea that CC-99677 may be effective in inflammatory diseases, such as AS, for which biologics against TNF and IL-17 have shown efficacy and are approved therapeutic agents [41].

The expression of TNF and other cytokines is controlled at the post-transcriptional level by the p38/MK2 pathway via TTP [17]. MK2 phosphorylates TTP at S52 and S178 after LPS stimulation and increases its stability [20]. As expected, MK2 inhibition resulted in reduced TTP phosphorylation, as reflected by reduced TTP abundance, in LPS-stimulated macrophages. Unphosphorylated TTP recruits deadenylases to ARE-containing mRNAs [19]. Indeed, actinomycin D treatment in our study demonstrated that CC-99677-mediated inhibition of TTP phosphorylation results in increased decay of cytokine mRNAs. These results support the mechanism of CC-99677 as an MK2 inhibitor that blocks cytokine production through stimulation of TTP-mediated ARE-containing cytokine mRNA decay. Future studies will explore the effect of CC-99677 on TTP-mediated translational repression.

Tachyphylaxis has been a consistent finding in clinical trials of multiple p38 inhibitors. Initial reductions in inflammatory markers were not sustained despite continuous dosing with p38 inhibitors [5, 7, 9, 10]. We generated a novel in vitro model of tachyphylaxis mimicking continuous exposure in an in vitro setting in THP-1 cells. This model was employed to determine the potential for tachyphylaxis with CC-99677 and to compare with p38 inhibitors. CC-99677, but not the p38 inhibitors BMS-582949, BIRB-796, and SCIO-469, consistently inhibited TNF secretion in THP-1 cells over the 14-day assay, suggesting feedback pathways may exist that prevent p38, but not MK2 inhibitors, from demonstrating sustained inhibition. While the mechanism of this difference is not yet understood, prior studies in THP-1 cells, macrophages, and synoviocytes have demonstrated the activation of c-Jun N-terminal kinase (JNK) upon treatment with the p38 inhibitors SB203580 and VX-745, but not with the MK2 inhibitor PH089 [12, 13]. In addition, p38 inhibition has been reported to disrupt the negative feedback by DUSP1, a phosphatase that is activated by p38 phosphorylation and dephosphorylates JNK [42]. p38 inhibitors are also reported to increase the activation of JNK by preventing the phosphorylation of TAK1 binding proteins (TAB1) and prolonging TAK1 activity [43]. Thus, tachyphylaxis observed with p38 inhibitors may be attributed to the disruption of the negative feedback exerted by p38 in controlling inflammatory responses. Future studies will address the differential effects of MK2 inhibition on these feedback pathways.

CC-99677 was well-tolerated with a once-daily oral dosing regimen over 2 weeks in healthy adult volunteers, with all AEs being mild in nature and the majority not suspected of relationship to CC-99677; these observations are similar to the well-tolerated FIH profiles seen with other MK2 inhibitors [44–46]. Despite sustained covalent binding of MK2, multi-cytokine inhibition by CC-99677 does not appear to be a cause or effect of a decrease in peripheral blood WBC or WBC subsets. The lack of neutropenia following 2 weeks of dosing with CC-99677 is encouraging given the mechanism of CC-99677 in inhibiting GM-CSF, TNF, and IL-6 production, as GM-CSF is important in stem cell differentiation into neutrophils, and both TNF and IL-6 blockade are associated with dose-dependent neutropenia [47, 48]. Nonetheless, larger studies in patients will need to confirm the initial safety profile presented here; a phase II study has been initiated in patients with AS (NCT04947579) to measure the safety and efficacy.

As expected for an irreversible covalent inhibitor, MK2 occupancy by CC-99677 accumulated over time with daily dosing, reaching a dose-dependent steady state between days 5 and 8. Pharmacodynamic effects of CC-99677 showed a dose-dependent relationship with ex vivo cytokine inhibition. Of importance, CC-99677 inhibition of TNF was sustained throughout the 14-day treatment period, which contrasts with the reported loss of effect of a p38 inhibitor, BCT197, utilizing a similar assay and clinical study design. Of interest, another MK2 inhibitor, ATI-450 (formerly CDD-450), showed inhibition of LPS-induced TNF for up to 4 weeks after dosing in a mouse model of neonatal-onset multisystem inflammatory disease (NOMID), whereas the global p38α inhibitor CDD-111 (also known as SD-0006) did not [49]. Overall, these lines of evidence suggest that MK2 inhibition may bypass the feedback pathways, which, when elicited by p38 inhibition, result in the tachyphylaxis and transient efficacy that have impeded the clinical development of p38 inhibitors. However, clinical evaluation in longer studies in a disease setting is necessary to confirm these findings.

As the experiments and FIH study were in vitro and ex vivo assessments, these findings cannot be fully extrapolated to results in patients with endogenously elevated pro-inflammatory cytokine levels. Phase II and III studies in large patient cohorts will be needed to confirm the findings suggested by the FIH experience. Nevertheless, the effects of CC-99677 were consistent across different experimental conditions, and the in vitro and ex vivo assays were replicable.

While the period between the late 1990s and mid-2010s was dominated by a brave new world of biologic proteins targeting single cytokines or their related receptors, recent drug discovery and development in rheumatology, dermatology, and other inflammatory diseases have been marked by small-molecule, multi-cytokine inhibitors. These approaches may offer improved efficacy and convenience. Inhibition of the JAK-STAT pathway, for example, has emerged as a viable treatment regimen for several conditions including rheumatoid arthritis, psoriatic arthritis, and inflammatory bowel disease [50]. Promising phase III data are also emerging in AS [51]. However, some safety liabilities exist for JAK inhibitors, with an increased risk of serious infections and a higher incidence of venous thromboembolic events; therefore, alternatives are desired [52]. As prescribers and patients move toward a preference for safe and effective oral options as opposed to injectable or infusible treatments, once-daily, oral, multi-cytokine inhibition with CC-99677 as a monotherapy or in combination with other oral agents holds promise for an era of new treatment paradigms. The observed lack of tachyphylaxis in in vitro and ex vivo assays of human cells exposed to CC-99677, compared with disappointing earlier attempts to target the p38 axis [7, 53], are encouraging findings and suggest that MK2 inhibition may prevail where direct p38 inhibition has historically failed.

## Conclusions

The results of the FIH study, as well as characterization of CC-99677 in vitro and ex vivo, show that inhibition of MK2 potently reduces inflammatory cytokines and chemokines relevant to the pathophysiology of AS. The CC-99677 safety and tolerability profile, linear PK profiles, and a high degree of target engagement resulting in sustained inhibition of inflammatory disease-relevant cytokines ex vivo suggest that further investigation of the therapeutic potential of CC-99677 in future clinical studies is worthwhile.

## Supplementary Information

**Additional file 1: Table S1**. Demographics. **Figure S1**. Multiple daily doses of CC-99677 were well tolerated in healthy volunteers. Proportion of active CC-99677 (*n* = 28) or placebo (*n* = 9) subjects with or without TEAEs (a). Number of TEAEs by dose level of CC-99677 and among placebo group (b). Frequency of TEAEs by MedDRA preferred term in the system organ classes in which at least 3 subjects receiving CC-99677 experienced an AE (c). The MedDRA system organ classes in which these AEs are grouped are: “nervous system disorders” (*n* = 5 active subjects), “gastrointestinal disorders” (*n* = 5 active subjects), and “musculoskeletal and connective tissue disorders” (*n* = 3 active subjects). Proportion of subjects with TEAEs by suspected relationship to CC-99677 by blinded investigator (*n* = 13 active subjects experiencing AEs) (d). TEAE treatment-emergent adverse event, MedDRA Medical Dictionary for Regulatory Activities.

## Data Availability

Bristol Myers Squibb’s policy on data sharing may be found at https://www.bms.com/researchers-and-partners/independent-research/data-sharing-request-process.html.

## Abbreviations

AE: Adverse event
ANC: Absolute neutrophil count
ANOVA: Analysis of variance
APC: Antigen-presenting cells
ARE: AU-rich elements
AS: Ankylosing spondylitis
*C*_max_: Peak concentration
CRP: C-reactive protein
DMSO: Dimethyl sulfoxide
DUSP1: Dual-specificity phosphatase 1
ELISA: Enzyme-linked immunosorbent assay
FBS: Fetal bovine serum
GM-CSF: Granulocyte-macrophage colony-stimulating factor
HuR: Human antigen R
FIH: First-in-human
IFN*γ*: Interferon gamma
IL: Interleukin
JNK: Jun N-terminal kinase
LPS: Lipopolysaccharide
MAPK: Mitogen-activated protein kinase
MAD: Multiple ascending dose
MCP-1: Monocyte chemoattractant protein-1
MIP: Macrophage inflammatory protein
MKK4/7: MAPK kinase 4/7
MK2: Mitogen-activated protein kinase-activated protein kinase-2
PBMC: Peripheral blood mononuclear cell
PD: Pharmacodynamics
PK: Pharmacokinetics
PBS: Phosphate-buffered saline
SD: Standard deviation
SEB: Staphylococcal enterotoxin-B
SMaSh: Streptavidin mass shift
TAB1: TAK1-binding proteins
TEAE: Treatment-emergent adverse events
TTP: Tristetraprolin
TNF: Tumor necrosis factor
UTR: Untranslated regions
WBC: White blood cell
