# Supplementary material for: CC-99677, a novel, oral, selective covalent MK2 inhibitor, sustainably reduces pro-inflammatory cytokine production
Source: Arthritis Res Ther. 2022 Aug 18;24:199. doi: 10.1186/s13075-022-02850-6 (PMC9386913; doi:10.1186/s13075-022-02850-6)

**Table S1. Demographics**

|  | **CC-99677  (*n* = 28)** | **Placebo (*n* = 9)** | **Total (*N* = 37)** |
| --- | --- | --- | --- |
| Male, *n* (%) | 18 (64.3) | 7 (77.8) | 25 (67.6) |
| Female, *n* (%) | 10 (35.7) | 2 (22.2) | 12 (32.4) |
| White, *n* (%) | 25 (89.3) | 8 (88.9) | 33 (89.2) |
| Age (years) |  |  |  |
| Mean (min, max) | 35.2 (21–55) | 30.8 (20–48) | 34.1 (20–55) |
| Height (cm) |  |  |  |
| Mean (min, max) | 174.3 (153–193) | 176.8 (162–186) | 174.9 (153–193) |
| Weight (kg) |  |  |  |
| Mean (min, max) | 79.66 (55.4–104.6) | 81.98 (59.2–115.0) | 80.22 (55.4–115.0) |
| BMI (kg/m^2^) |  |  |  |
| Mean (min, max) | 26.07 (20.0–32.7) | 26.09 (21.0–33.2) | 26.08 (20.0–33.2) |

# Figure S1. Multiple daily doses of CC-99677 were well tolerated in healthy volunteers. Proportion of active CC-99677 (*n* = 28) or placebo (*n* = 9) subjects with or without TEAEs (a). Number of TEAEs by dose level of CC-99677 and among placebo group (b). Frequency of TEAEs by MedDRA preferred term in the system organ classes in which at least 3 subjects receiving CC-99677 experienced an AE (c). The MedDRA system organ classes in which these AEs are grouped are: “nervous system disorders” (*n* = 5 active subjects), “gastrointestinal disorders” (*n* = 5 active subjects), and “musculoskeletal and connective tissue disorders” (*n* = 3 active subjects). Proportion of subjects with TEAEs by suspected relationship to CC-99677 by blinded investigator (*n* = 13 active subjects experiencing AEs) (d). *TEAE* treatment-emergent adverse event, *MedDRA* Medical Dictionary for Regulatory Activities

#
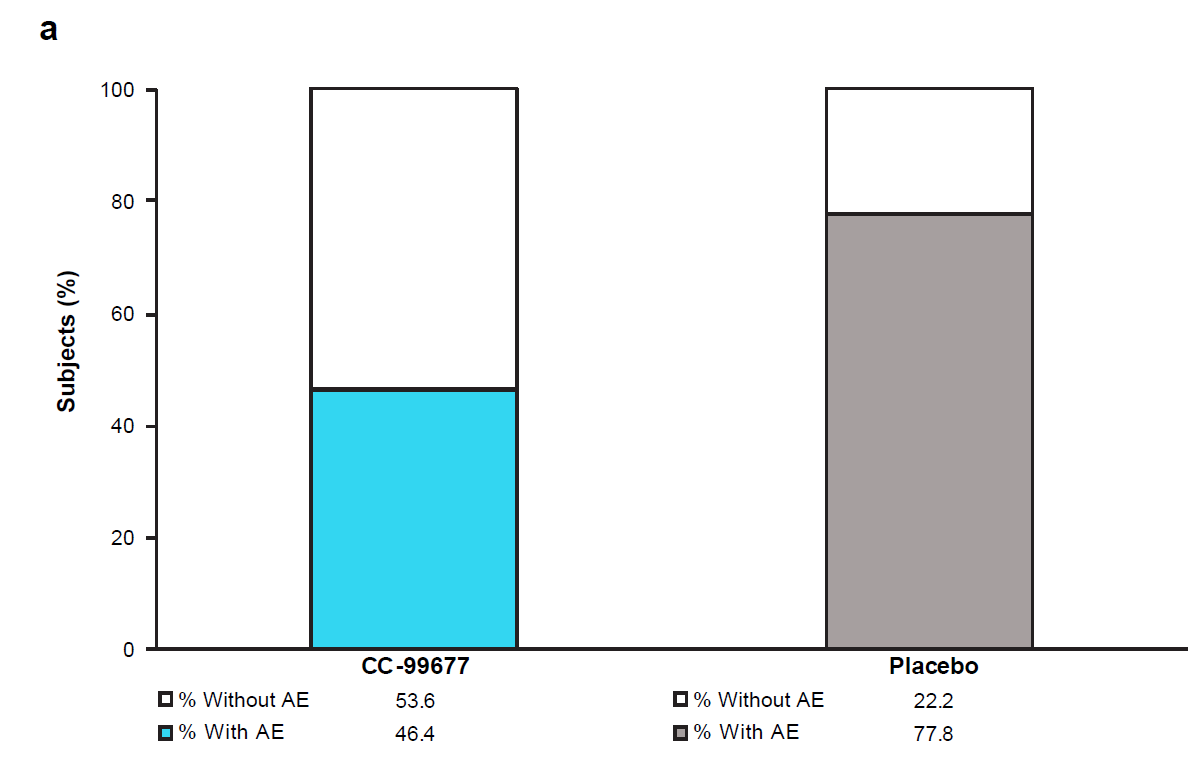


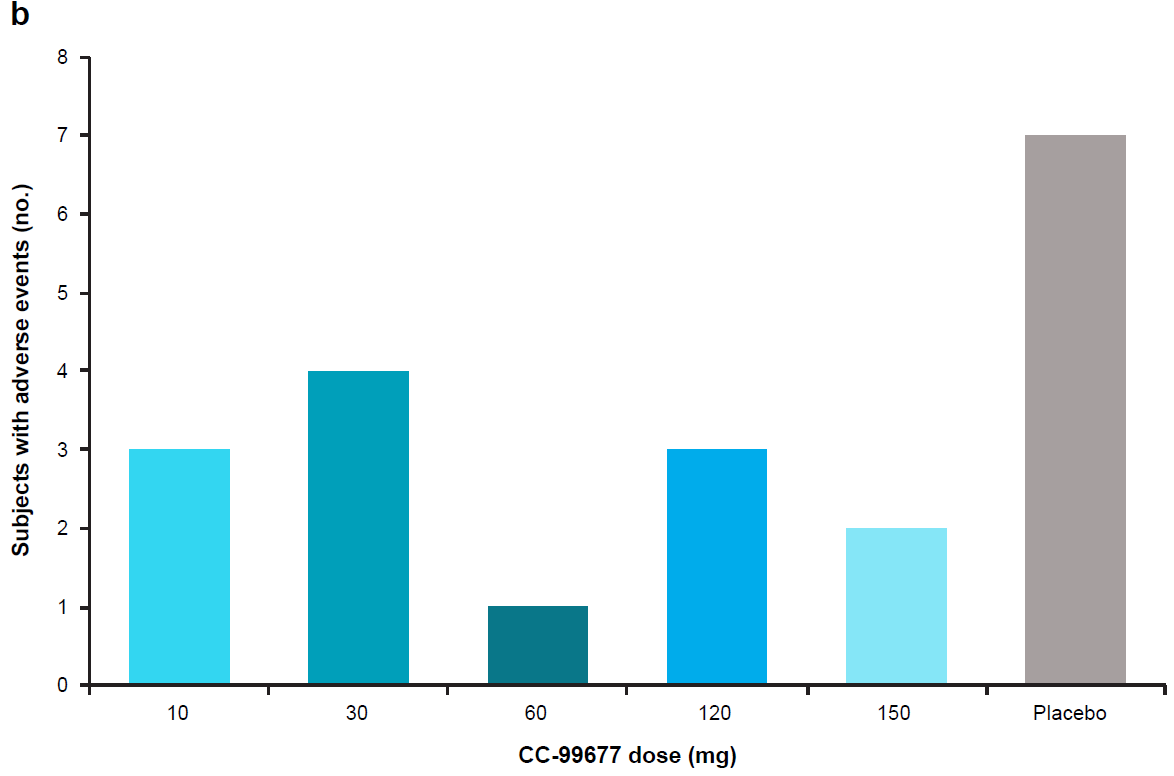


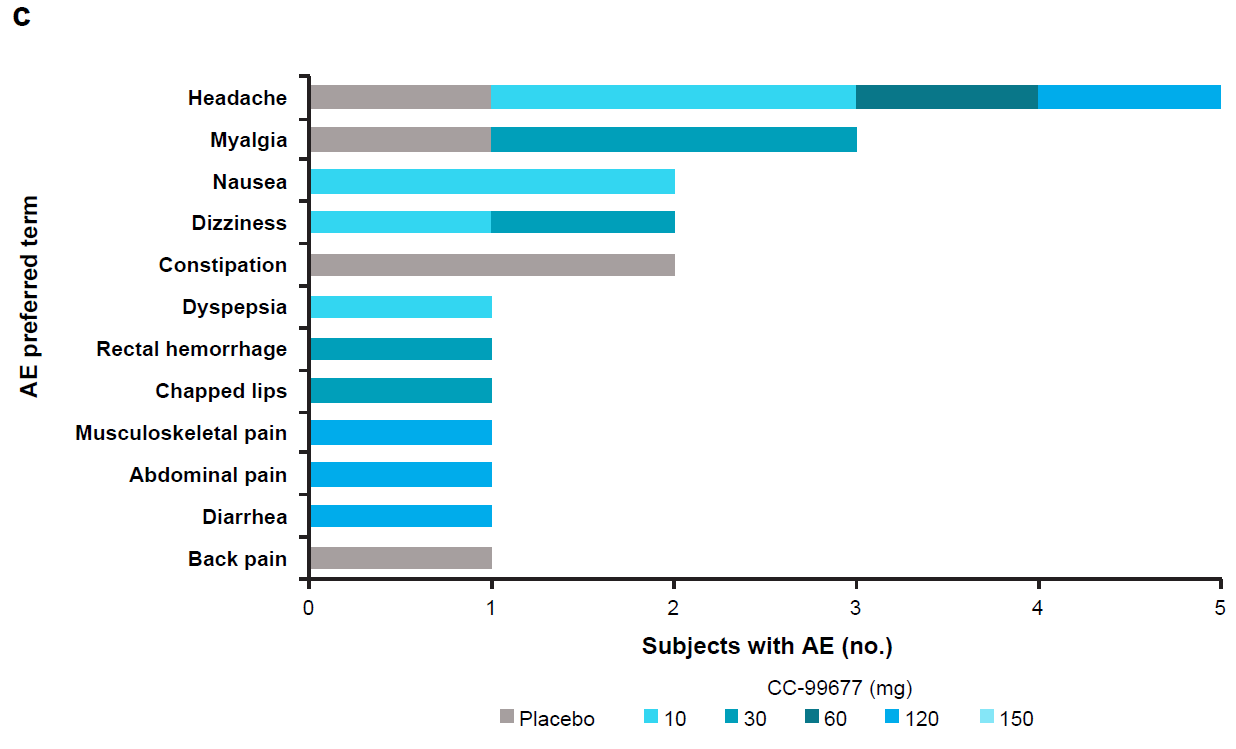


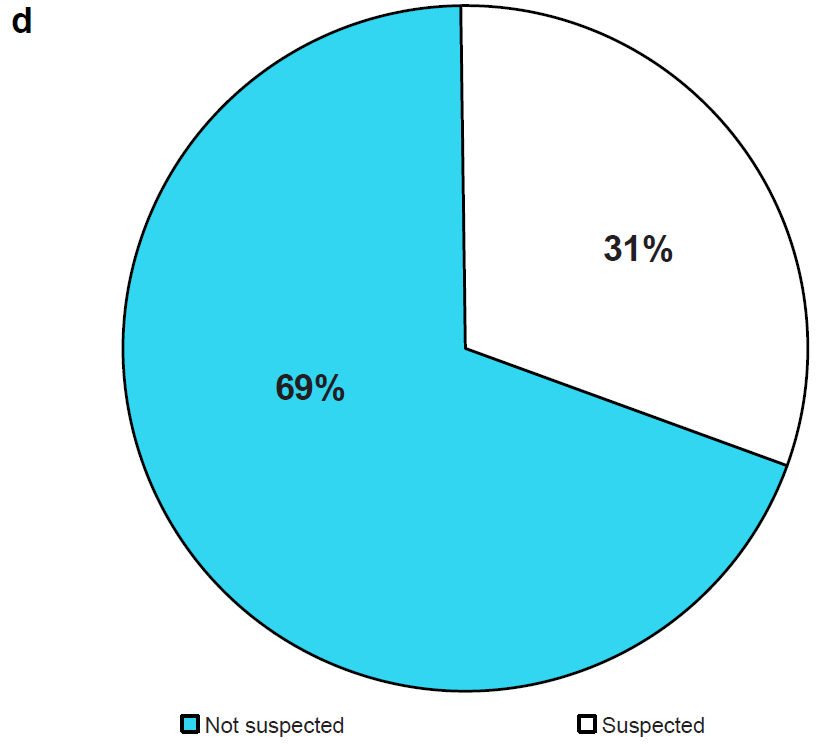

Supplement: Supplementary file 1 — Additional file 1: Table S1. Demographics. Figure S1. Multiple daily doses of CC-99677 were well tolerated in healthy volunteers. Proportion of active CC-99677 (n = 28) or placebo (n = 9) subjects with or without TEAEs (a). Number of TEAEs by dose level of CC-99677 and among placebo group (b). Frequency of TEAEs by MedDRA preferred term in the system organ classes in which at least 3 subjects receiving CC-99677 experienced an AE (c). The MedDRA system organ classes in which these AEs are grouped are: “nervous system disorders” (n = 5 active subjects), “gastrointestinal disorders” (n = 5 active subjects), and “musculoskeletal and connective tissue disorders” (n = 3 active subjects). Proportion of subjects with TEAEs by suspected relationship to CC-99677 by blinded investigator (n = 13 active subjects experiencing AEs) (d). TEAE treatment-emergent adverse event, MedDRA Medical Dictionary for Regulatory Activities. [file 13075_2022_2850_MOESM1_ESM.docx]
